# Supplementary material for: Immune Regulation, but Not Antibacterial Activity, Is a Crucial Function of Hepcidins in Resistance against Pathogenic Bacteria in Nile Tilapia (Oreochromis niloticus Linn.)
Source: Biomolecules. 2020 Jul 31;10(8):1132. doi: 10.3390/biom10081132 (PMC7464455; doi:10.3390/biom10081132)
Supplement: Supplementary file 1 [file biomolecules-10-01132-s001.pdf]

Table and figure legends

Table S1. Pairwise homology analysis of the nucleotide sequences of the *On*-Hep1 and hepcidin genes found in other vertebrates. Blue and green colors indicate identity and similarity, respectively.

Table S2. Similarity and identity of *On*-Hep1 and hepcidin genes found in other vertebrates available in the GenBank database using Blastx programs.

Table S3. Primers used in this study.

Figure S1. Phylogenetic analysis of hepcidin proteins in Nile tilapia (*On*-Hep1 and *On*-Hep2) and other vertebrates with 1,000 bootstrapping values. All amino acid sequences of organisms were obtained from the GenBank database with the indicated scientific names and accession numbers. Scale bar indicates genetic variation.

Figure S2. Overexpression analysis of rPro*On*-Hep1. SDS-PAGE was performed to detect the overexpression of Pro*On*-Hep1 in *E. coli* BL21 cells. Lane M: protein marker, 1: BL21, 2: BL21+1 mM IPTG, 3: Pro*On*-Hep1, 4: Pro*On*-Hep1+1 mM IPTG **(A)**. SDS-PAGE showing the production of Pro*On*-Hep1 protein in *E. coli* BL21 cells. Lane M: protein marker, 1: pellet of Pro*On*-Hep1+1 mM IPTG after sonication, 2: supernatant of Pro*On*-Hep1+1 mM IPTG after sonication **(B)**. SDS-PAGE showing the purification of Pro*On*-Hep1 protein from *E. coli* BL21 cells. Lane M: protein marker and lanes 1-13: purified Pro*On*-Hep1+1 mM IPTG obtained from fractions 1-13, respectively **(C)**.

Table S1. Phanaram *et al.* (2020)

	1	2	3	4	5	6	7	8	9	10	11	12	13	14	15	16	17	18	19	20	21	22	23	24	25	26	27	28	29	30	31	32	33
1. *On*-Hep 1 (*FF280957)		67.4	67.4	66.0	97.4	50.7	52.7	86.8	50.5	71.7	67.7	58.7	47.6	71.3	90.5	71.8	65.2	50.9	64.9	64.7	58.2	54.2	58.6	88.6	64.1	54.5	53.5	52.0	51.6	45.8	43.1	50.5	50.0
2. *On*-Hep 2 (AY725227)	68.1		96.3	73.3	68.8	43.9	53.0	67.1	51.8	83.5	59.1	47.8	51.9	76.7	69.5	85.2	49.5	62.9	68.2	67.9	54.5	49.6	55.5	67.0	48.7	53.3	47.6	49.8	49.8	42.4	45.1	51.1	49.6
3. *Oreochromis mossambicus* TH 1-5 (Huang *et al*., 2007)	68.1	96.3		72.9	68.1	42.1	53.3	67.1	52.0	81.5	58.4	47.8	52.1	74.8	69.2	83.1	49.5	61.4	67.5	66.3	53.1	49.1	55.0	67.0	48.7	53.5	47.8	49.8	49.8	44.6	46.1	50.2	49.8
4. *Oreochromis mossambicus* TH 2-2 (Huang *et al*., 2007)	68.1	75.0	74.5		67.0	48.1	50.6	64.9	46.2	74.8	58.1	46.3	55.0	68.6	66.0	71.5	50.2	54.4	61.3	60.1	51.9	48.3	54.8	63.5	49.6	50.5	50.2	51.1	51.4	44.6	41.9	50.7	47.9
5. *Oreochromis mossambicus* TH 2-3 (Huang *et al*., 2007)	97.4	69.6	68.9	69.2		50.4	52.4	85.0	50.2	72.4	65.3	58.3	47.3	72.0	89.4	73.3	64.8	51.3	65.9	65.5	56.8	53.8	60.2	86.8	63.7	54.1	52.7	48.4	48.0	43.6	43.8	48.5	48.6
6. *Salmo salar* (AF542965)	50.9	43.9	43.1	48.1	50.5		59.1	50.4	56.9	44.8	46.3	66.0	60.6	42.5	50.4	44.1	67.5	54.0	39.6	43.2	48.1	60.6	48.9	50.7	67.0	61.9	53.2	44.7	46.0	56.5	52.1	42.6	46.7
7. *Acanthopagrus schlegelii* (AY669376)	52.7	54.2	53.9	52.3	52.4	60.9		52.4	75.4	58.6	43.3	63.6	67.5	58.4	52.0	56.2	69.0	76.1	46.1	44.2	41.8	53.1	44.8	51.3	70.0	72.2	48.9	47.5	47.9	52.4	52.8	45.6	47.4
8. *Pagrus auriga* (AB440775)	86.8	68.1	68.1	66.3	85.0	50.5	52.4		49.5	71.7	68.2	64.1	48.0	71.8	93.0	72.5	67.4	50.9	67.4	67.2	58.5	53.6	58.4	92.3	64.5	55.2	52.5	51.3	50.7	44.3	42.1	49.6	53.3
9. *Dicentrarchus labrax* (DQ131605)	50.5	54.2	53.9	48.8	50.2	60.7	77.7	49.8		51.6	44.1	64.6	62.1	54.6	52.4	54.2	70.4	72.9	44.3	45.2	41.8	53.1	46.4	50.9	70.4	70.3	50.4	46.3	45.9	46.6	51.5	42.2	42.1
10. *Oplegnathus fasciatus* (EU809940)	73.3	83.5	82.4	75.7	74.0	44.9	59.9	73.3	53.9		59.1	48.0	52.4	82.1	73.5	89.1	52.0	64.4	70.7	70.0	53.6	48.5	55.7	72.4	50.4	54.2	48.9	51.1	51.1	42.5	41.9	51.4	51.6
11. *Gadus morhua* (EU334514)	67.7	59.3	58.6	58.2	65.3	46.8	43.4	68.7	44.1	59.9		48.5	42.4	57.2	68.0	58.2	47.8	42.8	57.9	53.8	59.5	51.9	56.9	68.4	48.1	45.2	47.8	47.8	47.5	44.5	40.8	50.7	52.7
12. *Paralichthys olivaceus* 1 (AY533022)	59.3	50.0	49.4	48.4	59.0	67.0	65.5	64.8	66.5	50.2	49.5		61.5	47.3	63.8	51.1	85.9	67.5	44.9	45.0	49.0	57.6	46.1	63.0	83.7	69.5	55.0	45.9	43.3	52.5	52.9	45.6	44.4
13. *Paralichthys olivaceus* 2 (AY533023)	47.6	51.9	52.1	55.0	47.3	62.8	67.5	48.0	67.0	52.4	42.4	61.5		50.0	49.8	51.7	65.2	69.6	46.4	46.1	40.2	51.3	45.1	49.1	65.0	68.4	46.0	44.3	43.9	48.2	49.2	40.2	44.6
14. *Sparus aurata* (AM749960)	71.8	77.5	75.7	71.2	72.5	43.4	58.4	71.8	55.8	83.9	57.6	48.3	50.2		72.9	80.1	50.9	55.4	67.4	68.5	53.9	52.2	54.2	73.6	51.3	54.1	50.2	51.8	50.2	42.2	42.9	50.4	54.3
15. *Micropterus salmoides* 1 (EU502749)	90.5	71.1	70.7	68.1	89.4	50.5	52.0	93.0	52.4	75.1	68.0	64.5	49.8	72.9		74.7	74.0	53.5	67.8	68.7	58.7	54.5	57.7	94.5	67.8	56.4	53.5	52.2	52.0	44.5	42.7	48.2	53.3
16. *Micropterus salmoides* 2 (EU502750)	71.8	85.2	83.1	73.2	73.3	44.1	57.5	72.5	56.7	89.1	58.2	52.9	51.7	80.1	74.7		53.1	72.4	69.6	68.5	54.5	48.1	54.8	74.4	53.0	54.6	50.2	51.8	52.2	45.2	45.2	48.7	50.9
17. *Micropterus dolomieu* 1 (EU502751)	65.2	52.3	51.7	53.1	64.8	67.5	69.0	67.4	70.4	54.3	47.8	87.2	65.5	52.1	74.0	55.6		71.9	46.4	46.5	49.5	59.3	48.6	68.9	91.6	75.2	55.3	47.1	44.6	52.2	50.2	44.4	45.8
18. *Micropterus dolomieu* 2 (EU502752)	50.9	62.9	61.4	55.0	51.3	56.5	76.1	50.9	77.5	64.4	42.8	67.5	69.6	55.4	53.5	72.4	71.9		47.4	46.3	40.1	52.2	45.8	52.7	72.0	72.4	50.2	45.8	45.8	53.5	51.5	45.0	44.7
19. *Epinephelus coioides* 1 (GU391241)	66.3	70.0	69.3	62.9	67.4	40.4	46.4	67.4	45.3	72.3	58.2	46.4	46.4	67.4	68.5	70.4	47.9	47.9		84.6	52.2	46.7	52.1	67.0	46.9	47.1	47.2	46.2	47.2	39.6	40.7	45.1	47.7
20. *Epinephelus coioides* 2 (GU391242)	65.2	70.5	68.5	64.0	65.9	43.2	44.7	67.4	46.2	71.5	54.2	47.3	46.6	70.0	69.2	70.1	48.5	47.3	84.6		54.9	49.8	55.1	67.8	46.2	45.9	45.0	49.3	48.9	40.9	40.4	49.5	48.7
21. *Ictalurus furcatus* (AY834210)	59.5	54.6	53.3	51.9	57.4	48.1	42.6	59.1	42.3	53.6	61.3	49.5	40.2	55.0	60.1	54.6	50.2	40.2	52.2	55.3		74.6	61.9	59.5	50.5	43.0	52.7	51.9	51.9	43.3	41.9	51.4	52.0
22. *Ictalurus punctatus* (AY834211)	54.9	50.4	49.1	48.4	54.6	60.6	54.8	54.6	53.8	49.1	51.9	58.4	52.0	52.8	55.3	48.3	60.6	52.9	47.9	50.8	74.6		56.9	54.7	62.2	54.1	56.0	51.4	49.8	52.3	50.4	51.0	49.8
23. *Danio rerio* (AY363452)	59.4	56.9	57.6	55.4	62.0	48.9	45.3	59.4	47.1	56.9	57.2	46.7	45.3	56.2	58.7	56.2	49.3	46.0	54.0	56.9	61.9	56.9		59.4	50.2	48.4	51.4	50.2	52.3	43.9	40.6	51.4	50.5
24. *Alphestes immaculatus* (HQ541866)	88.6	67.8	67.8	65.6	86.8	50.9	51.3	92.3	51.3	74.0	68.4	63.7	49.1	73.6	94.5	74.4	68.9	52.7	67.0	68.5	61.5	54.9	60.5		65.9	56.3	52.2	52.0	50.0	44.7	42.0	48.4	52.1
25. *Lates calcarifer* 1 (JF767495)	64.1	50.4	49.8	51.9	63.7	67.0	71.0	64.5	71.5	52.1	48.1	85.0	65.0	52.4	67.8	54.8	91.6	72.0	47.9	47.7	50.5	64.7	50.4	65.9		74.6	55.1	47.2	46.6	52.2	53.5	44.4	46.1
26. *Lates calcarifer* 2 (JF767496)	55.7	57.6	57.7	54.7	55.3	62.2	72.2	56.4	70.3	58.1	46.5	69.9	68.4	56.6	57.9	58.6	75.6	72.7	49.4	49.2	44.3	57.0	48.6	57.5	74.6		50.4	45.9	43.8	48.3	48.8	46.2	44.6
27. *Crocodylus siamensis* (FJ968771)	53.8	48.9	49.1	50.8	53.1	53.9	50.0	53.1	51.3	49.4	47.8	55.2	47.0	52.1	53.8	51.7	56.5	50.4	47.6	45.8	53.3	57.0	52.2	52.4	56.1	52.2		51.2	51.6	54.8	57.0	52.0	54.3
28. *Mus musculus* 1 (NM_032541)	53.1	52.7	52.1	54.7	48.7	44.8	48.4	52.4	47.2	52.8	47.8	46.4	44.4	53.2	53.1	54.8	48.0	46.0	47.2	51.5	52.2	51.6	50.4	53.1	47.2	47.2	51.6		93.7	52.8	49.2	66.3	69.8
29. *Mus musculus* 2 (AY232841)	52.4	52.7	52.1	55.0	48.0	46.0	48.8	51.6	46.8	52.8	47.5	43.3	44.0	52.4	53.5	54.8	45.6	46.0	47.6	51.1	51.9	50.0	52.9	50.9	46.8	44.4	52.0	93.7		51.2	47.2	64.3	66.7
30. *Canis lupus familiaris* (AY772532)	45.8	42.4	44.6	44.6	43.6	59.2	55.3	44.3	47.3	42.7	44.8	53.0	48.9	42.3	44.7	45.6	53.7	56.0	39.7	41.7	43.3	52.5	44.2	44.7	53.5	48.8	54.8	52.8	51.2		77.7	57.4	57.9
31. *Sus scrofa* (NM_214117)	43.2	45.1	46.1	41.9	44.0	52.4	53.3	42.1	53.2	41.9	41.1	55.0	50.8	43.1	42.9	45.6	50.7	52.4	40.8	41.3	41.9	51.1	40.6	42.1	54.0	49.8	57.0	49.2	47.2	77.7		61.0	55.7
32. *Bubalus bubalis* (EU399814)	50.9	52.3	50.9	52.7	48.7	42.6	46.2	50.5	43.4	53.2	51.9	46.2	40.2	52.8	49.1	50.6	45.0	45.0	46.1	51.1	52.2	53.0	52.5	49.8	44.6	47.0	52.6	66.3	64.3	59.4	61.0		78.4
33. *Homo sapiens* (NM_021175)	50.5	53.0	51.3	49.6	49.1	47.1	49.8	56.0	43.9	52.8	52.9	44.7	45.1	56.6	56.0	53.3	46.7	45.1	49.4	50.4	52.6	51.0	51.1	53.5	46.7	47.5	54.9	70.6	67.5	59.2	55.7	78.4	

Table S2. Phanaram *et al.* (2020)

Common name	Scientific name	GenBank accession no.	Similarity	Identity
**Nile tilapia (*On*-Hep1)**	*Oreochromis niloticus*	*FF280957	-	-
**Nile tilapia (*On*-Hep2)**	*Oreochromis niloticus*	AAU25840	68.1	67.4
**Mozambique tilapia**	*Oreochromis mossambicus* TH 1-5	Huang *et al.* (2007)	68.1	67.4
	Oreochromis mossambicus TH 2-2	Huang *et al.* (2007)	68.1	66.0
	Oreochromis mossambicus TH 2-3	Huang *et al.* (2007)	97.4	97.4
**Atlantic salmon**	*Salmo salar*	AAO85553	50.9	50.7
**Japanese black porgy**	*Acanthopagrus schlegelii*	AAU00801	52.7	52.7
**Redbanded seabream**	*Pagrus auriga*	BAH03285	86.8	86.8
**European seabass**	*Dicentrarchus labrax*	AAZ85124	50.5	50.5
**Striped beakfish**	*Oplegnathus fasciatus*	ACF49394	73.3	71.7
**Atlantic cod**	*Gadus morhua*	ACA42769	67.7	67.7
**Olive flounder**	*Paralichthys olivaceus* 1	AAT01563	59.3	58.7
	Paralichthys olivaceus 2	AAT01564	47.6	47.6
**Gilthead seabream**	*Sparus aurata*	CAO78619	71.8	71.3
**Largemouth black bass**	*Micropterus salmoides* 1	ACD13023	90.5	905
	Micropterus salmoides 2	ACD13024	71.8	71.8
**Smallmouth bass**	*Micropterus dolomieu* 1	ACD13025	65.2	65.2
	Micropterus dolomieu 2	ACD13026	50.9	50.9
**Orange-spotted grouper**	*Epinephelus coioides* 1	ADC93804	66.3	64.9
	Epinephelus coioides 2	ADC93805	65.2	64.7
**Blue catfish**	*Ictalurus furcatus*	AAX39714	59.5	58.2
**Channel catfish**	*Ictalurus punctatus*	AAX39715	54.9	54.2
**Zebrafish**	*Danio rerio*	AAR18592	59.4	58.6
**Pacific mutton hamlet**	*Alphestes immaculatus*	AER00227	88.6	88.6
**Asian sea bass**	*Lates calcarifer* 1	AEO51036	64.1	64.1
	Lates calcarifer 2	AEO51037	55.7	54.5
**Siamese crocodile**	*Crocodylus siamensis*	ADA68357	53.8	53.5
**Common house mouse**	*Mus musculus* 1	NP_115930	53.1	52.0
	Mus musculus 2	AAO73588	52.4	51.6
**Dog**	*Canis lupus familiaris*	AAV40979	45.8	45.8
**Wild boar**	*Sus scrofa*	NP_999282	43.2	43.1
**Asian water buffalo**	*Bubalus bubalis*	ABY81280	50.9	50.5
**Human**	*Homo sapiens*	NP_066998	50.5	50.0
**Mozambique tilapia (beta-actin)**	*Oreochromis mossambicus*	BAA90688	-	-

Table S3. Phanaram *et al.* (2020)

Gene	Primer names	Primer sequence 5'⟶3'	Amplicon size (bp)	Accession number	Purposes
**1. Interleukin-1β**	*On*-IL1 F	GTGCTGAGCACAGAATTCCAGGAT	166	XM_019365841	qRT-PCR analysis
	On-IL1 R	GAAGAACCAAGCTCCTCTTTTGGC			
**2. Interleukin-8 (CXC1)**	*On*-IL8 1F	TGTCTGTGTCACCGTGTCAGGAAT	151	FF279523	qRT-PCR analysis
	On-IL8 1R	CCTTCAGCTCAGGGTTCAAGCAAT			
**Interleukin-8 (CXC2)**	*On*-IL8 2F	CAAGCAGGACAACAGTGTCTGTGT	102	FF279523	qRT-PCR analysis
	On-IL8 2R	GTTGCAGAATTTGGTTGCTGGGTAG			
**3. CC chemokine1**	*On*-CC1 F	ACAGAGCCGATCTTGGGTTACTTG	228	KJ535436	qRT-PCR analysis
	On-CC1 R	TGAAGGAGAGGCGGTGGATGTTAT			
**4. CC chemokine2**	*On*-CC2 F	TGGGTTCGTGCCAAGATTGTTGCA	120	KJ535435	qRT-PCR analysis
	On-CC2 R	TGAAGGAGAGGCGGTGGATGTTAT			
**5. Hepcidin1**	*On*-Hep1 F	AGGGAGCAATGACACTCCAGTTGT	172	FF280957	qRT-PCR analysis
	On-Hep1 R	GAATCCTCAGAACCTGCAGCAGAA			
	Pro *On*-Hep1 F	AACATATGGAGAGCTCTGCCATCCCATTT	227	FF280957	Overexpression
	Pro *On*-Hep1 R	CTCGAGTCAGAACCTGCAGCAGAAGCC			
**6. Hepcidin2**	*On*-Hep2 F	GAAGACGTTCAGTGTTGCAGTTGC	169	AY725227	qRT-PCR analysis
	On-Hep2 R	CATCTTCCATGAGTCCACTGATGC			
**7. Transferrin**	*On*-Trans F	GGTGCTAACTACATGAGCATCGTC	148	DQ272465	qRT-PCR analysis
	On-Trans R	CGGTGTCATCAGACACACTGTTGA			
**8. β-actin**	*On*-β-actin F	ACAGGATGCAGAAGGAGATCACAG	155	KJ126772	qRT-PCR analysis
	On-β-actin R	GTACTCCTGCTTGCTGATCCACAT			

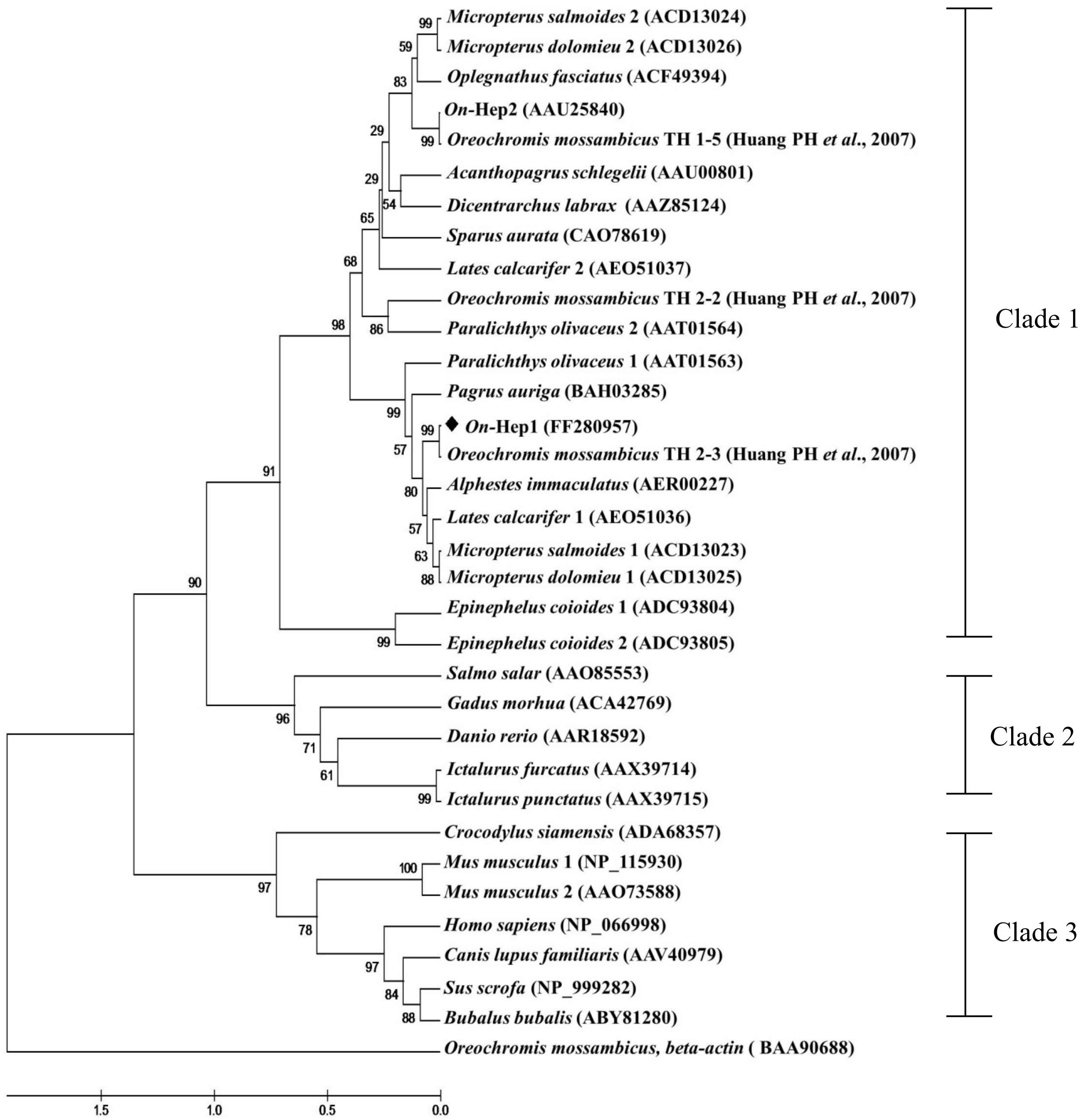

Figure S1. Phanaram *et al.* (2020)

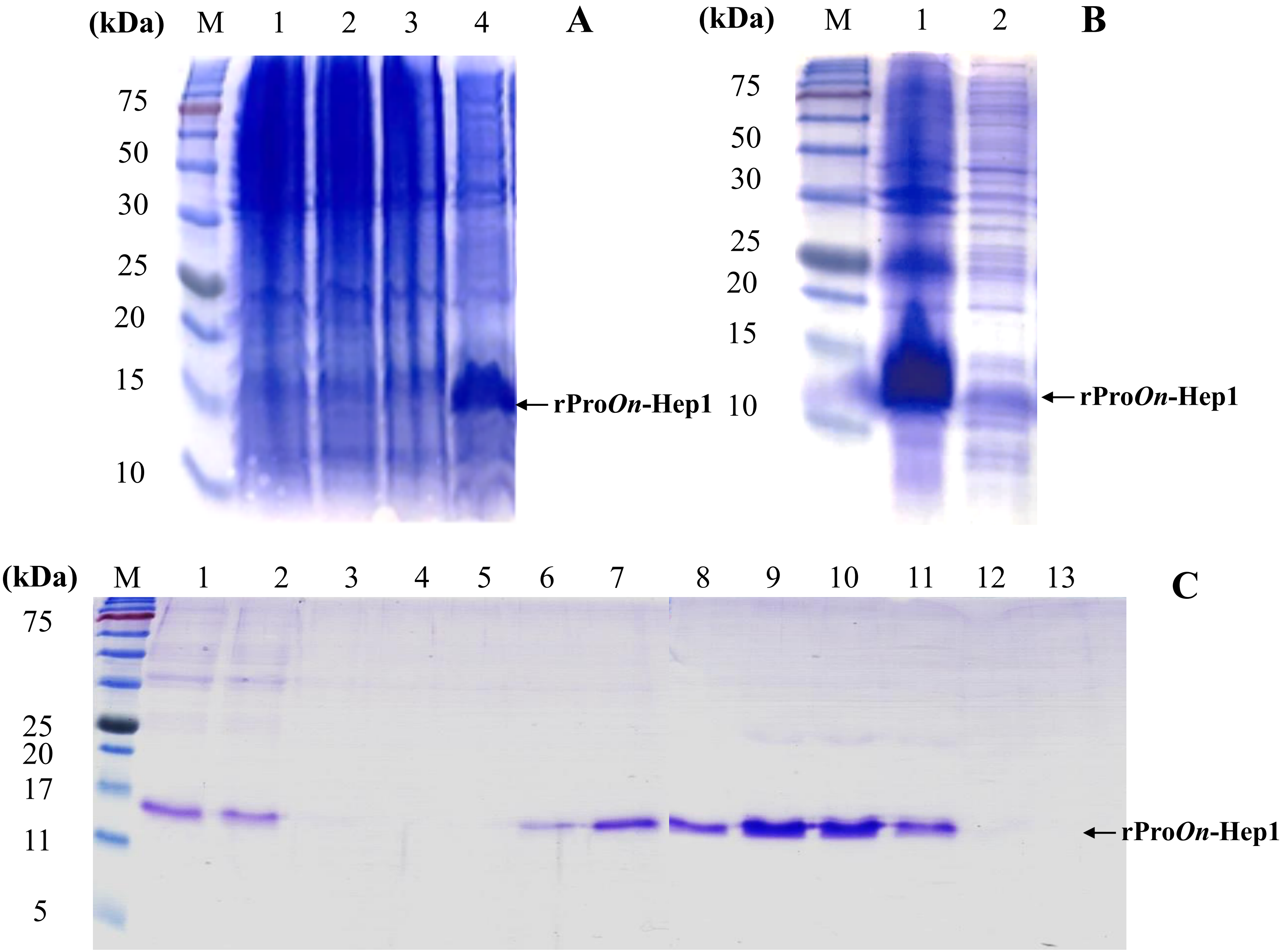

Fig S2. Phanaram *et al.* (2020)
